# Vectors and transmission dynamics for *Setaria tundra *(Filarioidea; Onchocercidae), a parasite of reindeer in Finland

**DOI:** 10.1186/1756-3305-2-3

**Published:** 2009-01-06

**Authors:** Sauli Laaksonen, Milla Solismaa, Raine Kortet, Jussi Kuusela, Antti Oksanen

**Affiliations:** 1 Fish and Wildlife Health Research Unit, Finnish Food Safety Authority Evira (FINPAR), P.O. Box 517, FI-90101 Oulu, Finland; 2 University of Oulu, Department of Biology, P.O. Box 3000, FI-90014 Oulu, Finland

## Abstract

**Background:**

Recent studies have revealed expansion by an array of Filarioid nematodes' into the northern boreal region of Finland. The vector-borne nematode, *Setaria tundra*, caused a serious disease outbreak in the Finnish reindeer population in 2003–05. The main aim of this study was to understand the outbreak dynamics and the rapid expansion of *S. tundra *in the sub arctic. We describe the vectors of *S. tundra*, and its development in vectors, for the first time. Finally we discuss the results in the context of the host-parasite ecology of *S. tundra *in Finland

**Results:**

Development of *S. tundra *to the infective stage occurs in mosquitoes, (genera *Aedes *and *Anopheles*). We consider *Aedes *spp. the most important vectors. The prevalence of *S. tundra *naturally infected mosquitoes from Finland varied from 0.5 to 2.5%. The rate of development in mosquitoes was temperature-dependent. Infective larvae were present approximately 14 days after a blood meal in mosquitoes maintained at room temperature (mean 21 C), but did not develop in mosquitoes maintained outside for 22 days at a mean temperature of 14.1 C. The third-stage (infective) larvae were elongated (mean length 1411 μm (SD 207), and width 28 μm (SD 2)). The anterior end was blunt, and bore two liplike structures, the posterior end slight tapering with a prominent terminal papilla. Infective larvae were distributed anteriorly in the insect's body, the highest abundance being 70 larvae in one mosquito. A questionnaire survey revealed that the peak activity of Culicidae in the reindeer herding areas of Finland was from the middle of June to the end of July and that warm summer weather was associated with reindeer flocking behaviour on mosquito-rich wetlands.

**Conclusion:**

In the present work, *S. tundra *vectors and larval development were identified and described for the first time. *Aedes *spp. mosquitoes likely serve as the most important and competent vectors for *S. tundra *in Finland. Warm summers apparently promote transmission and genesis of disease outbreaks by favouring the development of *S. tundra *in its mosquito vectors, by improving the development and longevity of mosquitoes, and finally by forcing the reindeer to flock on mosquito rich wetlands. Thus we predict that global climate change has the potential to promote the further emergence of Filarioid nematodes and the disease caused by them in subarctic regions.

## Background

There is recent evidence documenting the range expansion of parasites of domestic and free-ranging ungulates to subarctic areas including, in Finland, an array of vector-borne Filarioid nematodes and the diseases associated with them [1-3]. These findings are not surprising, since the potential impacts of global warming are predicted to include shifts in the spatial-temporal distribution of disease vectors, and hence the transmission dynamics of vector-borne diseases [4]. There is still a lack of basic knowledge, however, about how insect populations, their vector competence, and parasite-host ecology respond to these changes.

*Setaria tundra *(Filarioidea: Onchocercidae) was the causative agent of severe outbreaks of peritonitis in semi-domestic reindeer (*Rangifer tarandus tarandus*) in Finland in 1973 and in 2003–05, and in moose (European elk, *Alces alces*) in Lapland in 1989 [1]. The most recent outbreak started in 2003 in the southern part of the Finnish reindeer herding area and the focus of the outbreak has moved northwards approximately 100 km each year.

In this latest outbreak the prevalence and level of infection were very high in the calves, and caused substantial economic losses to the reindeer herders. Adult reindeer were suggested as the main source of infection for the calves [1,2], together with wild forest reindeer (*Rangifer tarandus fennicus*) and roe deer (*Capreolus capreolus*) [1,2].

The genus *Setaria *includes 43 species that are found in the abdominal cavities of artiodactyls. All these species produce microfilariae (mf) which are present in host blood where they are available to the arthropod vectors. The mf are taken up in the blood meal of the vector where they develop into the infective third larval stage. When the vector feeds again, the larvae break out and enter the tissue of the definitive host [5].

The life cycle of *S. tundra *in the Northern Europe is poorly understood. In California, the mosquito *Aedes sierrensis *serves as a vector for a related species, *Setaria yehi*, which parasitizes white-tailed deer (*Odocoileus virginianus*). In this host-parasite system, transmission is facilitated by the peak appearance of mosquitoes following the fawning season of the deer [6]. Other known insect vectors for *Setaria *spp are other *Aedes *spp. [7,8], *Anopheles *spp. [9-12], and horn flies, *Haematobia irritans *[13] and *H. stimulans *[13-15].

To date there is only scant information about the transmission of *S. tundra *other than the recent studies in Finland on the peritonitis outbreak in reindeer and mf burden in cervids [1,2]. The assumption that *S. tundra *is transmitted by haematophagous vectors is supported by the seasonal midsummer peak in *Setaria *microfilaria (Smf) density in reindeer [2], and by the well known attacks on herds of caribou/reindeer in the summer by massive swarms of blood-feeding insects [16].

The aims of the present study were: 1) to identify significant vectors for *S. tundra *in Finland; 2) to describe larval development in these vectors; and 3) to use this knowledge to explore basic features of parasite transmission, including outbreak dynamics and the rapid geographic expansion of *S. tundra *in Finland.

## Results

### Setaria tundra larvae in wild haematophagous insects

**Figure 1:**
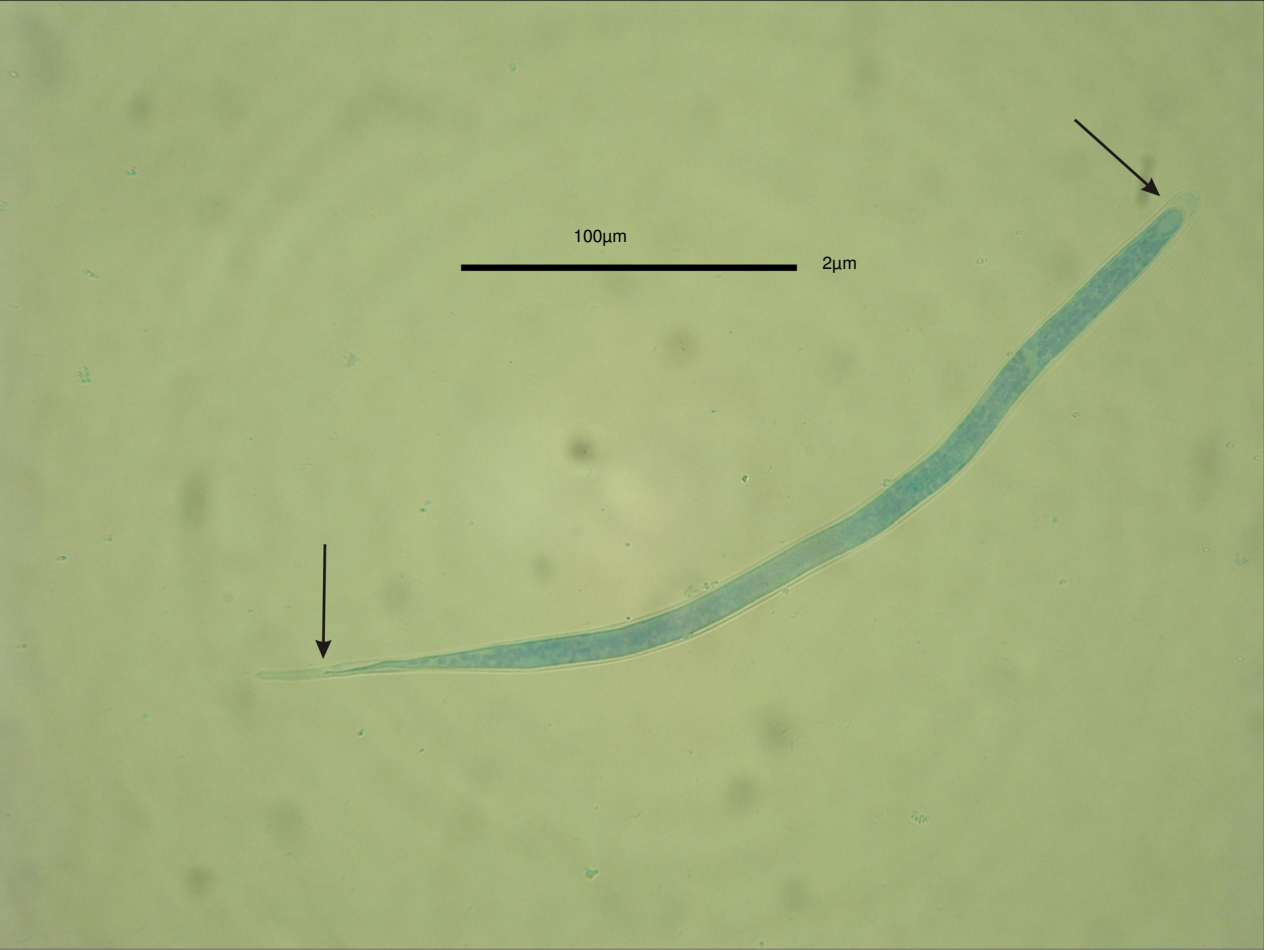
**Sheated (arrows) *S. tundra *microfilaria in the gut of *Aedes *sp. immediately after blood meal**.

Data for *S. tundra *occurrence in wild-netted *Aedes *spp. mosquitoes and in sympatric reindeer are presented in Table 1. Larvae of the parasite in different developmental stages ("sausages", L_2 _and L_3_) were detected in *Aedes communis, A. punctor, A. hexodontus, A. excrucians*. Oviposition status numbers was determined for 166 mosquitoes (*Aedes *spp.) dissected, of which 64 (38.5%) had laid eggs one or more times. All captured blackflies (Simulidae), biting midges (Culicoidea) and hornflies (*Hydrotea spp*.) were negative for developing *S. tundra *larvae, although Smf (Figure 1) were detected (1–800/insect) in the guts of these insects after a blood meal.

**Table 1:** Recovery of *Setaria tundra *larvae from *Aedes *spp. mosquitoes and of microfilariae from reindeer in six locations in Finland, summer 2004.

Place	Diss. mosq. (captured)	Inf. mosq.	Prevalence (%)	No of *S. tundra *larvae,(range)	smf-prev in reindeer	Collection time
Perho	200 (443)	1	0,5	8	15% ^1^	July 22^th^
Kuhmo	148 (148)	1	0,7	5	36% ^1^	July 19^th^
Kuusamo	210 (2061)	1	0,5	3	61% ^2^	July 3^th ^– August 28^th^
Sodankylä	200 (254)	4	2	3 (2–4)	10% ^2^	July 29^th^
Kaamanen	200 (286)	0	0	-	0% ^2^	July 28^th^
Oulu Zoo	200 (1680)	5	2,5	2 (1–3)	100% ^2^	June 10^th ^– August 20^th^
Tot	958 (4429)	12	1.2			

^1^Wild forest reindeer, ^2^Reindeer

All hibernating *Culiseta *and *Anopheles *spp. mosquitoes were nulliparous and none contained *S. tundra *larvae.

### The development of S. tundra in mosquitoes

#### Trial I: outdoor conditions

Species of *Aedes *included in the study were: *Aedes communis *(n = 14); *A. hexodontus *(n = 8); *A. punctor *(n = 4); and other unidentified *Aedes *spp. (n = 9). All 35 mosquitoes were nulliparous. The prevalence of *S. tundra *immediately following the blood meal was 57%, and the number of developing larvae in infected individuals varied from 1 to 25 (mean 7, SD 5.4). On day 1 after the blood meal, mf were already unsheathed (mean length 255.8 μm, SD 14.2, mean width 6 μm, SD 0.97), and were still in the abdomen. During day 2 the larvae migrated into the thorax (n = 10 mosquitoes). Larvae reached the sausage stage (late first-stage, mean length 122.5 μm, SD 14.4, mean width 16.6 μm, SD 2.2) on days 6 to 9 after the blood meal and remained in the late first-stage or early second stage after day 16 (mean length 185.6 μm, SD 18.6, mean width μm 29.9, SD 6.5). No third-stage infective larvae were observed up to day 22 after the blood meal, when the trial ended.

#### Trial II: laboratory conditions

Of the mosquitoes included in this trial, 97 belonged to genus *Aedes *and 7 to genus *Anopheles*, and all were nulliparous.

**Figure 2:**
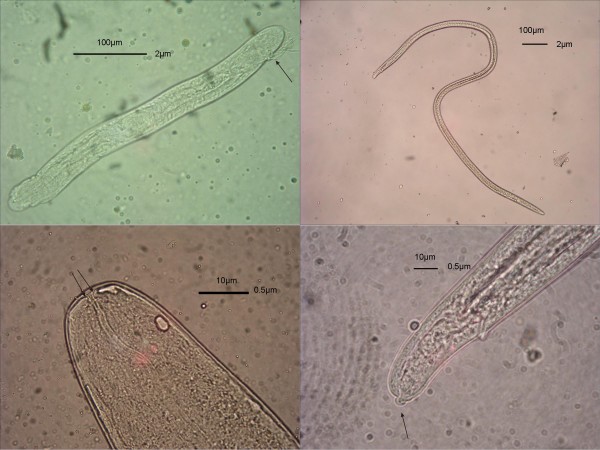
**The development of *S. tundra *larvae in *Aedes sp*. mosquito**. a. 2^nd ^stage larvae with visible anal plug (arrow). b. Elongated 3^rd ^stage larvae: The identification details of S. tundra infective 3^rd ^stage larvae in.; c. Blunt anterior end with two liplike stuctures (arrows). d. Tapering posterior end with prominent terminal (knoblike) papilla (arrow).

Following the blood meal, developing *S. tundra *larvae were observed in 40 of the *Aedes *spp. mosquitoes (prevalence 41%) and individual mosquitoes harboured 1 to 51 larvae (mean 8, SD 10.7). The development of *S. tundra *larvae in *Aedes *mosquitoes is presented in Table 2 and Figure 2. During day 1, the larvae unsheathed and migrated to the thoracic muscles. Larvae reached the sausage stage after days 4 to 6, and at this stage had a short, distinct tail and, in the more developed larvae, the mouth, oesophagus and intestine were quite distinct. After days 9 to 10 the larvae were slender and had approximately doubled in length. In these second-stage larvae, the anal plug was visible, but the tail had been lost during the first moult. At this stage, the lumen of the intestine and oesophagus were distinct. Finally, approximately two weeks after the blood meal, and following the second moult, infective elongated third-stage larvae were present. The anterior ends of these larvae were blunt, slightly tapering and bore two liplike structures, the posterior ends were slightly tapering from the anus with a prominent terminal (knoblike) papilla. The intestines were broad, round, and tubular and showed much variation. The cuticule had transverse striations.

**Table 2:** The development of *S. tundra *larvae in laboratory insectary conditions (21°C, SD 2.8, relative humidity 65%, SD12.4) in *Aedes *mosquitoes.

Days after blood meal	1	2–3	4–6	7–8	9–10	11–13	14–16	17–20	21–22
No	2	2	10	9	4	3	2	3	5
Mean larvae (SD) Range	1 -	1.5 (0.5) 1–2	2.8 (1.9) 1–7	3.3 (1.2) 1–5	11 (8.4) 3–24	8.4 (6.5) 3–21	5,5 (2.5) 3–8	28 (18) 8–51	17 (11,2) 6–34
Mean length (SD) Range	266 (9.3) 257–275	194 (19) 176–220	191 (42) 132–330	295 (60) 165–385	451 (99) 198–803	885 (388) 165–1210	1687 (104) 1540–1760	1333 (180) 935–1595	1504 (155) 1100–1760
Mean width (SD) Range	7.9 (1.1) 6.75–9	25 (4.9) 20–32	23 (4.4) 14–32	31 (5) 18–36	35 (2.5) 32–41	38 (6.2) 23–45	29 (2) 27–32	27,4 (1) 27–32	29 (2,3) 27–36
Development site	thorax	Thorax	thorax	thorax	thorax	thorax	thorax, abd, head	thorax, abd, head, prob, palps	Thorax, abd, head, prob, palps
Larvae tot.	2	3	28	26	43	30	11	83	84

Approximately 51% of the infective larvae were located in the thorax, 31% in the head or proboscis and 18% in the abdomen. Measurements of older *S. tundra *larvae in five *Aedes *specimens are presented in Table 3.

**Table 3:** . Details of *S. tundra *larvae obtained from five *Aedes *mosquitoes.

Species	Days after blood meal	Larvae (n)	Mean length, (SD), range	Mean width, SD, range	Development site (n)
*Aedes *sp.	1	2	broken	38.25 μm 36–40.5	thorax
*Aedes *sp.	2–3	15	1634 μm, (132), 1375–1870	32.7 μm (2.5), 31.5–40.5	thorax (13) proboscis (1) abdomen (1)
*Aedes *sp.	2–3	70	807 μm, (271), 418–1210	37.6, (2.2), 36–40.5	thorax (69) abdomen (1)
*Aedes *sp.	4–6	4	Broken	45 μm	thorax
*Aedes *sp.	7–8	20	1115 μm, (277.8), 855–1500	50.6 μm, (5.5), 45–59.4	thorax

The larvae were excluded from the development measurements because they were considered older, originating from previous blood meals.

Six of the seven *Anopheles *spp. collected contained *S. tundra *larvae. Three of the infected mosquitoes examined 6–7 days after the blood meal contained developing larvae (mean burden 2.7 (SD 1.2); mean length 253 μm (SD 60); mean width 29.3 μm (SD 5.5)). At each examination at days 9–10, 11–13 and 21–22 after the blood meal a single *Anopheles *mosquito was found infected and 4, 3 and 7 larvae, respectively, were recovered. Measurements of these larvae were: mean length 440 μm (SD 95.3), 231.5 μm (SD 9.2) and 1423 μm (SD 68.7); and mean width 36.6 μm (SD 0.97), 32.6 μm (SD 5.6) and 29.3 μm (SD 2.1), respectively.

Melanisation was recognized in 11 mosquitoes during the developmental studies. Melanised larvae were mostly microfilariae in the abdomen but more-developed and melanised larvae were also observed in the thorax.

### PCR studies

The 680 bp mtDNA sequence to the sequences from all 6 *S. tundra *larvae examined were identical to those from adult *S. tundra *parasitizing reindeer in northern Finland (GenBank DQ097309) [1]. The partial 18S ribosomal RNA gene sequences of the *S. tundra *larvae were also identical to those from *S. tundra *microfilaria from the blood of these reindeer (GenBank EF081341) [2].

### Questionnaire survey

**Figure 3:**
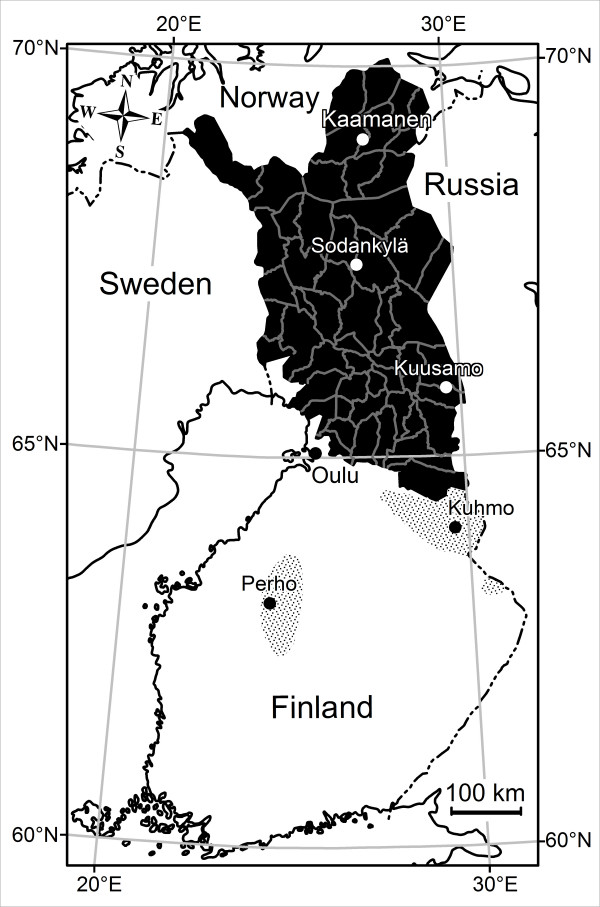
**Finnish reindeer herding area (black) divided in 56 reindeer herding cooperatives and two wild forest reindeer populations (shaded)**. The foci of *S. tundra *associated disease [1] and *S. tundra *microfilaremia in reindeer blood [2] was in 2003 in the area 1, and moved northwards during next two years into the area 3, while the area 4 remained free of infection.

**Figure 4:**
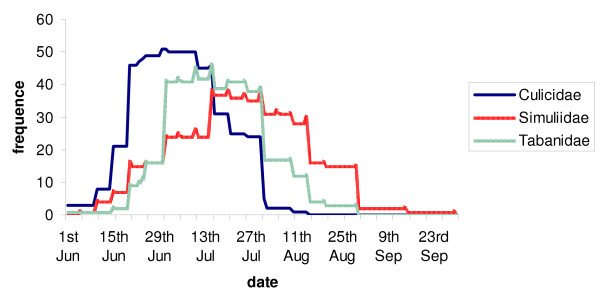
**The frequency and activity patterns of räkkä-insects (Culicidae, Simuliidae and Tabanidae) in the summer months in Lapland (data from the questionnaire)**.

All Chiefs of District of the 56 reindeer herding cooperatives in the Finnish reindeer herding area (Figure 3), responded to the questionnaire. The results indicate that overall harassment by flying mosquitoes during the summers of -2003, -2004 and -2005 was less severe than the average (for 2003, 69% of respondents, for 2004, 77%, and for 2005, 46%, respectively) or about average (29%, 22% and 52% or respondents, respectively) in the whole reindeer herding area. Respondents also reported that the peak activity of Culicidae occurs from mid-June to the end of July (Figure 4). Moreover, the mosquito feeding activity on reindeer was reported to be the highest in the evenings (37% of respondents) or at dawn (18%) and at dusk (10%) or if warm enough, at night (18%). According to 86% of the Chiefs, weather conditions and insect harassment alter reindeer behaviour so that during warm weather reindeer flock mostly in wetlands, swamps and riversides, and sometimes in forests (18% of respondents) in fells (14%) and different kinds of open expanses (16%). In cool summers, flocking behaviour is diminished. In areas of high *S. tundra *prevalence some Chiefs (11 of 56) reported on their own initiative that the prevalence of disease associated with *S. tundra *(peritonitis detected at slaughter) in reindeer herds varied depending on the site of herd's summer pasture.

## Discussion

This study has provided evidence that mosquitoes, particularly *Aedes *spp. and to a lesser extent *Anopheles *spp., have an important role in the transmission of *Setaria tundra *in the reindeer herding areas of Finland. The study has also provided information on the morphological changes in *S. tundra *larvae developing in mosquitoes, and on the distribution of these larvae in the vectors. It has also demonstrated that at a mean temperature of 21 C larvae develop to the infective third stage in approximately two weeks, while at a mean temperature of 14.1 C, development is not completed. Finally, questionnaire results suggest a linkage between levels of vector activity in the field and the transmission of *S. tundra *among reindeer.

The morphological changes, distributions and development rates of *S. tundra *in mosquitoes, observed in the present study are similar to those for other *Setaria *spp. described previously [5,9,11,17,18].

In the present study there was considerable variation in the morphology of the developing larval stages, as observed earlier for species of the Filarioidea developing in different vectors [19]. These variations might make the identification of the larvae difficult other than at the family level, and illustrate the role of PCR-based methods for species identification.

The vast majority of mosquitoes active in midsummer in Finland are species of *Aedes *[20]. Although *Anopheles *mosquitoes are also present, and can serve as vectors for *S. tundra*, their epidemiologic significance in Finland is likely limited because of their life cycle parameters and low numbers compared to *Aedes *spp. [20]. The role of *Anopheles *mosquitoes may be more important in more temperate areas or may increase in Finland during climate change. Our results suggest that *S. tundra *is not a very vector-specific parasite, and this may enhance its ability to expand its geographic range. Also, adult female *Aedes *spp. are vigorous round-the-clock feeders that can be infected with many infective *S. tundra *larvae (up to 70 in the present study). This high larval abundance may, however, decrease vector efficiency and increase vector mortality.

The transmission of *S. tundra *is highly dependent on the life span of the female mosquitoes, with survival of adult mosquitoes depending in part on both temperature and humidity [21]. There is unfortunately no information on the longevity of mosquito populations in Finland, although in the present study adult *Aedes *spp. survived approximately four weeks in a laboratory insectary at room temperature. In the present study, older females were a considerable part of the "wild" mosquito population. It is interesting that although the *S. tundra *outbreak in Sweden in 1973 was associated with the appearance of especially large numbers of mosquitoes [22], the longevity of the mosquitoes is also important.

The peak period of *S. tundra *microfilaremia in reindeer is from mid June to the end of August [2], which data from the questionnaire suggest co-incides with the peak activity of mosquitoes. Some estimates suggest that reindeer can be exposed to attacks of approximately 8000 mosquitoes/hour during the räkkä-time (the appearance of mass insect harassment) [23]. These peaks in microfilaremia and insect abundance and contact with reindeer occur just after the calving season, when the reindeer shed their winter hair leaving their skin relatively exposed to the attacks of mosquitoes. This synchrony likely promotes the transmission of *S. tundra *from adult carriers to calves [2].

In highly endemic areas, the prevalence of Filarioid infection rarely exceeds 1% of the total mosquito population [24]. In this study, *S. tundra *prevalence in different mosquito populations in endemic areas was 0.5–2.5%, the highest prevalence being around the experimental zoo, an urban park-like environment, on the outskirts of Oulu city.

In the present study, according to the questionnaire results, warm summers were associated with hundreds of reindeer flocking in dense herds in mosquito-rich wetlands. This behaviour may be explained by the availability of drinking water and fresh food plants or the benefits of the habitat for thermoregulation [16]. In these areas the microclimate is favourable for mosquitoes and it is possible that in these kinds of highly endemic areas, the prevalence and abundance of *S. tundra *in mosquitoes is very high. In a previous study in upper Lapland, the ancient destination of reindeer summer migration, neither peritonitis [1] nor mf [2], were found in reindeer, and there mosquitoes infected with *S. tundra *were not found. These observations suggest that the migrations of reindeer and the characteristics of reindeer pastures can affect the transmission dynamics of *S. tundra*. This may help explain the numerous discoveries by reindeer herders of great variation in *S. tundra *levels in reindeer herds originating from different summer pastures. In Uzbekistan, the highest prevalence of *S. labiatopapillosa *in cattle was found in irrigated, marshy and river catchment areas [25]. It is also important to note that wetlands constitute about 30 to 40% of the total land area of the *S. tundra *outbreak area but only about 12% of upper Lapland, [26] which is believed to be free of *S. tundra *[1,2].

## Conclusion

The present study has revealed the lack of basic knowledge of filarial larvae in their vectors in northern latitudes. In Finland, mosquitoes, especially *Aedes *spp., are efficient and likely the most important vectors of *S. tundra*. A key factor promoting the transmission of this parasite is warm ambient summer temperature. Within limits, this warmth improves mosquitoes' development, reproduction, longevity and feeding habits, as well as the larval development of *S. tundra*. Warm summers, which may become warmer as a consequence of climate change, also force the reindeer to flock and stay on mosquito-rich wetlands, behaviour which might increase the infection pressure. It is likely that climate change [27] favours the northward expansion of Filarioid nematodes, which might then become an even greater threat to arctic ungulate populations. The current study has provided baseline information that will improve understanding of the ecology and dynamics of *S. tundra *and of disease outbreaks associated with the parasite. The information could also be useful in predicting and preventing these outbreaks.

## Methods

### Identification of significant vectors for S. tundra

To estimate the prevalence of *S. tundra *larvae in arthropod populations in different *S. tundra *endemic areas, insect samples were collected from various locations (Figure 3). The samples were taken by netting insects attacking the experimental reindeer or humans on pastures with high reindeer density. Simultaneously S. *tundra *infection rates in the reindeer were monitored from blood samples (see [2]). Individual insects were randomly selected from the samples and examined for *S. tundra *larvae. Collected and examined mosquitoes are presented in Table 1. Other examined insects and collection times were: Perho; 13 black flies (July 22^th^), Kuhmo; 111 black flies and 6 *Hydrotea *spp. (July 19^th^), Kuusamo; 192 black flies and 88 *Hydrotea *spp. (July 3^th ^– August 28^th^), Sodankylä; 305 black flies (July 29^th^), Kaamanen; 129 black flies (July 28^th^), Oulu University Experimental Zoo; 55 black flies, 1267 biting midges and 119 *Hydrotea *spp. (June 10^th ^– August 20^th^) (Figure 3).

To examine the possibility of *S. tundra *larvae surviving in mosquitoes over the winter, hibernating and newly emerged *Anopheles *spp. (n = 25) and *Culiseta *spp. (n = 95, *Culiseta alaskaensis *34, *Culiseta bergrothi *61) mosquitoes were collected between April 24^th ^and May22^nd^, 2005 from winter caves in pastures in Kuusamo grazed by reindeer with high *S. tundra *prevalence [1,2].

Field-netted insects were killed using ether and stored frozen before dissection. Haematophagous insects were identified to genus and when possible to species. During dissection, the mosquitoes were divided into three parts (head, thorax and abdomen) which were examined using a stereomicroscope and mounted separately in a drop of *Aedes*-ringer [28] on a slide. A coverslip was then added and the specimen examined under a compound microscope at 40 ×. Oviposition status of the mosquitoes was determined according to Detinova [29]. All developing larvae found in the study were photographed and their length and width measured. The specimens were identified to family according to Bain and Chabaud [30].

### PCR studies

Six larvae were collected from six mosquitoes: one from a wild *Aedes *mosquito from Kuusamo and five from *Aedes *mosquitoes from the second development trial. Mosquitoes were digested in 10 μl of a solution containing 0,45% Tween 20 (Merck, Germany) and 0.45% Igepal CA-630 (Sigma-Aldrich, Germany), PCR-buffer (10 mM Tris-HCl, 1.5 mM MgCl2, 50 mM KCl and 0.1% Triton X-100) (Finnzymes, Finland) and 500 μg/ml Proteinase K (Finnzymes, Finland). Samples were incubated at 65°C for 30 min followed by 10 min at 95°C. For one PCR reaction, 2–5 μl of digestion solution was used. The presence of *S. tundra *was demonstrated by comparing mitochondrial DNA sequences with all known sequences of *S. tundra*, as well as other Filarioidea species. The primers StCoI 616L and StCoI 1321H were used to amplify part of CoxI gene. The specific *S. tundra *primers and PCR conditions were as previously described [1,2]. Because many sequences for 18S rRNA genes of different Filarioidea species are available in GenBank, we decided to amplify also that gene. Two new 18S rRNA gene primers had been developed earlier for the identification of microfilariae in cervid blood [2]. All known suitable 18S rRNA sequences of Filarioidea in GenBank was used as templates. The sequences of these primers are: 18S_F2 5' CCGCGGTAATTCCAGCTC and 18S_R1 5' CCTACGGAAACCTTGTTACGAC. These primers allow the amplification of partial 18S rRNA genes (1.2 kp).

### Larval development of S. tundra in mosquitoes

Two trials exploring the development of *S. tundra *larvae in mosquitoes were conducted at the Oulu University Experimental Zoo. The zoo is located on the university campus within the city of Oulu, but outside the outside the reindeer herding area, and there are no contacts between the zoo reindeer and other reindeer or roe deer. The eight reindeer in the zoo, which served as the source of the blood meal for the mosquitoes at the start of the trials, were naturally infected with *S. tundra *and their Smf densities in the blood were continuously monitored (see [2]). Mosquitoes which were seen to feed on the reindeer were captured by a paper cup pooter, upholstered with cotton wool, on the vertical surfaces of the reindeer feeding shelter.

#### Trial I, outdoor conditions

Mosquitoes (n = 34) were trapped on June 17^th ^and 18^th^, 2004. Mean Smf density in the blood from the zoo reindeer was 1007/ml (SD 661, range 109–1800). The trapped mosquitoes were individually maintained outdoors (average daily mean temperature 14.1°C, SD 2.6 and relative humidity 70.1%, SD 6.6) in small plastic vials permeable to air. The mosquitoes were provided with daily 10% glucose solution and water provided ad lib. The liquids were served via soaked blotting paper and cotton wool which were daily renewed.

#### Trial II, laboratory conditions

The mosquitoes (n = 104, 97 *Aedes*. spp, and 7 *Anopheles *spp.) were netted at the zoo on August 3^rd^, 2004. On this day, the mean Smf density in the blood of the reindeer varied from 0 to 2900/ml (mean 1183, SD 1090). The trapped mosquitoes were maintained under constant insectary conditions in the laboratory (21°C, SD 2.8, relative humidity 65%, SD 12.4) and fed as in trial I.

Mosquitoes were periodically killed in freezer (-80°C, 30 s.) before examination. For data treatment, they were divided into categories 1, 2–3, 4–6, 7–8, 9–10, 11–13, 14–16, 17–20 and 21–22 days after blood meal.

Five of the *Aedes *mosquitoes trapped contained *S. tundra *in advanced stages of development, indicating that they had fed on infected reindeer some days before capture. These were excluded from the developmental studies, together with the seven infected *Anopheles *spp. mosquitoes captured.

### Questionnaire survey

To assess the timing and severity of the mass appearance of blood sucking insects during the *S. tundra *outbreak in the summers 2003, 2004 and 2005, and also the behavioural response of reindeer to the resulting insect harassment and to the prevailing weather conditions, in 2006 a questionnaire was administered to all Chiefs of District of the 56 reindeer herding cooperatives in the Finnish reindeer herding area. Respondents were also asked about any experiences concerning outbreaks of disease in the area associated with *S. tundra*. The questionnaire was mailed to participants, and in some cases completed by telephone.

## Competing interests

The authors declare that they have no competing interests.

## Authors' contributions

SL conceived and designed the study and participated in the realization of the study and trials. SL also drafted the manuscript. MS participated in the design and performed the arthropod dissection and larvae extraction. RK participated in the manuscript writing. JK carried out the molecular genetic studies and drafted the manuscript. SN and SS carried out the morphological studies and drafted the manuscript. AO participated in the design and coordination of the study and was active in writing. All authors involved in the analysis of the data, gave their views, read and approved the final manuscript.
